# Comparison of Different Additive Manufacturing Methods for 316L Stainless Steel

**DOI:** 10.3390/ma14216504

**Published:** 2021-10-29

**Authors:** Javier Bedmar, Ainhoa Riquelme, Pilar Rodrigo, Belen Torres, Joaquin Rams

**Affiliations:** Department of Applied Mathematics, Materials Science and Engineering and Electronics Technology, Escuela Superior de Ciencias Experimentales y Tecnología (ESCET), Universidad Rey Juan Carlos, Mostoles, 28933 Madrid, Spain; javier.bedmar@urjc.es (J.B.); ainhoa.riquelme.aguado@urjc.es (A.R.); pilar.rodrigo@urjc.es (P.R.); belen.torres@urjc.es (B.T.)

**Keywords:** selective laser melting, direct laser deposition, additive manufacturing, 316L, mechanical properties

## Abstract

In additive manufacturing (AM), the technology and processing parameters are key elements that determine the characteristics of samples for a given material. To distinguish the effects of these variables, we used the same AISI 316L stainless steel powder with different AM techniques. The techniques used are the most relevant ones in the AM of metals, i.e., direct laser deposition (DLD) with a high-power diode laser and selective laser melting (SLM) using a fiber laser and a novel CO_2_ laser, a novel technique that has not yet been reported with this material. The microstructure of all samples showed austenitic and ferritic phases, which were coarser with the DLD technique than for the two SLM ones. The hardness of the fiber laser SLM samples was the greatest, but its bending strength was lower. In SLM with CO_2_ laser pieces, the porosity and lack of melting reduced the fracture strain, but the strength was greater than in the fiber laser SLM samples under certain build-up strategies. Specimens manufactured using DLD showed a higher fracture strain than the rest, while maintaining high strength values. In all the cases, crack surfaces were observed and the fracture mechanisms were determined. The processing conditions were compared using a normalized parameters methodology, which has also been used to explain the observed microstructures.

## 1. Introduction

Additive manufacturing (AM) of metals comprises a set of techniques that consists in the fabrication of pieces, layer by layer, from a 3D design. AM has several advantages over other manufacturing processes, as it allows fabricating components with complex geometries [1], which are not within the reach of conventional techniques [2]. Moreover, an increase in complexity of AM pieces does not increase manufacturing costs, and may even reduce them. This fact brings the industry closer to manufacturing topologically optimized pieces, which, together with the absence of molds, reduces the costs of fabrication and material waste. Another benefit of AM is its ability to personalize products in different fields, like prototyping in the automotive industry, the aerospace industry, or in jewelry and biomedical industries, in which the manufacturing of unique pieces for specific cases is required [3]. On the other hand, AM needs a high initial investment, since metal printers are more expensive than polymer printers due to their complex technology and energy requirement; they are also more sensitive to the metallic powder used.

In addition, 3D printed metal pieces have characteristic defects that are not present with other manufacturing techniques. The main problems are the porosity induced by gaps left due to the powder shape and flow, and the formation of residual stress and large grain growth caused by the different consecutive processes of melting, remelting, and heat treatment, caused by layer by layer deposition. Another disadvantage, which is common in all AM processes, is the presence of a layered structure in the entire manufactured piece due to the system of fabrication. These effects can reduce certain properties of the material, like its strength or corrosion resistance [4,5].

There are several kinds of metal 3D printing, and these techniques can be divided into two large groups: powder bed fusion and directed energy deposition (DED). In PBF an energy source is used to melt the metal, layer by layer, in a bed of powder. Each time a layer is melted or sintered, another layer of powder is deposited on the bed and the platform is lowered to melt it. This type of 3D printing can use a laser or an electron beam to melt the metal. This technique needs gas to avoid the oxidation of metal, such as argon or nitrogen, or a vacuum. In DED techniques the melting and deposition of powder occur at the same time using, usually, through the use of a laser. The deposition of the fused material is on a platform, which can be moved on some axes. This technique also needs an inert gas to avoid oxidation. In this study, three techniques have been used: two PBF processes, selective laser melting (SLM) using a fiber laser and a CO_2_ laser, and a DED method, direct laser deposition (DLD) using a high-power diode laser (HPDL) [3,6].

In SLM, a laser is used to melt the powder in a bed, performing fusion and refusion of the powder. SLM allows the production of near-net-shape parts with relatively full densities and good mechanical properties. This technique has a number of parameters to control, such as the scan speed, build direction, or infill orientation [7].

DLD is a DED technique that allows manufacturing in two axes (longitudinal and height), which does not allow complex geometries (on contrary to the previous case), but its speed of fabrication is greater due to the fact that the deposition and the melting of the powder happen at the same time. Although this method cannot build very complex geometries, it can make pieces with a low porosity [8,9,10].

A laser is an indispensable tool in this kind of manufacture. In this study, three different types of lasers were analyzed: diode laser, fiber laser, and CO_2_ laser. Diode lasers have many advantages, particularly their low cost, reduced maintenance, low power consumption, homogeneous intensity distribution in the beam, and high absorptivity for metals. They have a beam with a low optical quality, which limits the focusing ability of the laser, preventing the manufacturing of highly detailed structures [11].

In fiber lasers, the gain medium is a rare-earth-doped optical fiber. These are versatile and show a high quantum efficiency (~94%). Their emission is near infrared and, thanks to being a fiber-based gain medium and its optical components having a high beam quality, showing robustness against environmental disturbances, as well as system compactness. However, its performance is limited by nonlinear effects in the gain medium, and by polarization instabilities [12]. Despite this, the good beam quality and the high absorbance of metals due to its wavelength have made these lasers the most commonly used ones in SLM equipment.

CO_2_ lasers have been the most used industrial lasers for years. In these lasers, a gas is electrically pumped by a current (DC or AC) to induce population inversion for lasing. CO_2_ lasers provide a high efficiency (20%), an output power from 0.1 to 20 kW, and are relatively simple, reliable, and have a low-cost. Due to this, CO_2_ lasers are used in different applications: cutting, welding, marking, surface modification, etc. The main disadvantage of these lasers is that they emit light at a 10.6 μm wavelength, which is more complex to control, resulting in beams with greater divergence and a greater laser focus size [13]. They also have a low absorptivity by many metals, such as aluminum [12].

Out of the different metals that can be used in AM, 316L austenitic stainless steel is one of the most used as it has high corrosion resistance [14,15] and good mechanical properties [16,17]. The 316L is appropriate for AM because it is not affected by the presence of oxygen very much, has a low thermal conductivity and a high melting point and, in addition, it has high absorptivity in infrared, which makes it adequate for most AM equipment. Chen et al., 2017 [18] additively manufactured 316L components using DLD and, later, Gong et al., 2019 [19] fabricated 316L components using SLM, both groups obtained similar results with respect to the tensile properties of the AM components, comparable to wrought 316L properties. Some researchers have compared the properties of AM 316L pieces with samples fabricated using conventional techniques, such as melting, hot pressing, and casting, and found that similar or better properties can be obtained through additive manufacturing. Bartolomeu et al., 2017 [20] achieved similar tribological behavior for 316L SLM components for several conventionally manufactured samples. Other researchers found that several properties improve in AM samples. AM 316L has a higher yield strength than conventional ones, while maintaining a high ductility. Barkia et al., 2020 [21] observed that a hierarchical and dissimilar AM microstructure results in an excellent strength, while the low stacking fault energy induces the presence of high deformation twinning, which produces the high ductility of the AM 316L. However, other studies found that the corrosion behavior of AM 316L can be improved [22].

Different AM techniques provide strong differences in the microstructure of samples, which explains the differences in the observed properties. The DLD method is characterized by the slow cooling of samples due to the large volume of material that is molten for the deposition of each layer during manufacturing. As a result, large grains are usually observed, although they are usually smaller than in conventional processing techniques. Mixtures of equiaxial grains in the central part of molten pools with directional grains at the edges of the pools have been observed. This allows DLD 316L steels to usually show a higher hardness and elastic yield compared to conventional ones, but with values that are lower than those of other AM techniques. In the same sense, these materials have a relatively high fracture strain [23]. On the other hand, SLM 316L steels show a much finer microstructure with a clear directional morphology. The microstructure arises from the buildup of molten pools that cross one another with the manufacturing angle (in many cases 67° between layers); as such, from the top, the microstructure has intersecting molten pools, and from the sides, they have fish-scales shapes. Equiaxial grains appear within the molten pools and directional columnar ones appear at the boundaries. Regardless of all this, epitaxial grains usually appear across the different molten pools, resulting, in many cases, as a deleterious microstructure for mechanical and corrosion behavior [24].

A comparison of different AM techniques must consider all the parameters that change between cases to have a rigorous comparison. For powder bed melting AM techniques, Thomas et al. [25] derived a series of normalized parameters that allow comparing different manufacturing conditions. The main one is E*_min_ (Equation (1)), which is the normalized minimum heat input per unit volume needed to melt the added powder, and E*_0_ is:

E*_min_ = [*A q* / (*2 v l r_B_*)] [1/*ρ Cp* (*T_m_*−*T*_0_)] (1)

E*_0_ = E*_min_ r_B_ / *h* (2)

E*_min_ depends on the surface absorptivity, *A*, laser power *q*, laser scanning speed *v*, layer height *l*, laser spot radius *r_B_*, density of material *ρ*, specific heat *Cp*, and the difference between melting temperature *T_m_* and temperature of powder used *T*_0_. During heating, hatch spacing (*h*) is also important as it indicates the amount of material that is molten several times during manufacturing. These normalized parameters have been used to compare the processing windows of manufactured samples. In the case of the DLD samples, these parameters cannot be used in a straightforward way, as some of the molten material is lost and because the melting of the material beneath the deposited layer is much greater.

The properties of the manufactured samples are influenced by the parameters of manufacturing [26], but AM using SLM CO_2_ lasers has not been studied. The processing window of fiber laser SLM has been studied in several works [27,28], and the parameters considered have been with E*_0_ values between 2 and 8. In addition, the inverse of the relative layer height (spacing hatch divided by layer height) used for 316L is about 0.25, but values as high as 2 are used with other materials.

In the case of DLD, the processing window has not been established with the same methodology, but using these normalized parameters provides values in the range of 1–3 for E*_min_; larger values lead to melting of the manufactured structures and lower ones lead to very porous structures [23]. In DLD, due to the thickness of the walls, the inverse of the relative layer height is very high, as minimal overlapping is used between adjacent layers, so E*_0_ cannot be established for some conditions.

In this work, for the first time, the microstructure and mechanical properties of the 316L stainless steel made using SLM with a CO_2_ laser are studied. In addition, the microstructure of these samples is compared with those manufactured using SLM with a fiber laser and DLD using a HPDL. Different build-up orientations and scanning strategies are compared and hardness, bending resistance, and fractography are studied in the different samples manufactured.

## 2. Materials and Methods

**Figure 1:**
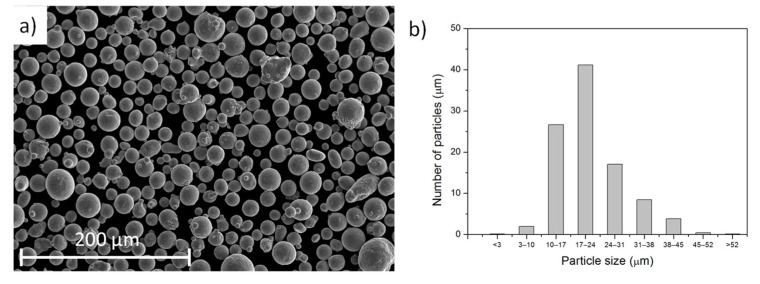
(**a**) SEM image of the 316L powder used; (**b**) histogram of particles size.

The AISI 316L stainless steel powder used (LPW Technology) (Figure 1a) had the following nominal composition (in wt %): chromium (16–18), nickel (10–14), molybdenum (2–3), manganese (≤2), silicon (≤1), nitrogen (≤0.1), oxygen (≤0.1), phosphorus (≤0.045), carbon (≤0.03), sulphur (≤0.03), and Fe (rest). The morphology of the particles was spherical (Figure 1a) with a particle size in the range of 20 to 50 microns, although most of the particles were between 15–35 microns, as shown in Figure 1b. The pieces were fabricated using three different additive manufacturing processes: DLD with a diode laser, and two SLM systems, one with a fiber laser and the other with a CO_2_ laser. The characteristics of each process are summarized in Table 1.

In the case of the DLD process, the used AM equipment consisted of a continuous wave diode laser (ROFIN DL013S, Hamburg, Germany) with a wavelength of 940 nm and a coaxial nozzle for the powder (IWS COAX 8 from Fraunhofer) [29]. The 316L powder was sprayed coaxially with the laser beam through a nozzle and the powder focus was 13 mm below the nozzle tip. Argon was used as a carrier gas at 4.5 atm pressure and at a 0.05 L·s^−1^ flow rate. The laser was placed on a 6-axis robot ABB and a hot plate connected to a temperature control system was used as the substrate support. To avoid excessive heat buildup, the laser power was linearly reduced from 600 W to 100 W during the deposition process. Previous studies determined that samples are not capable of dissipating heat provided by the laser, which increases the temperature of the previously deposited layers and causes the melting of the built structures. As buildup progresses, the temperature of the layers is greater than that at the initial point, so the required input heat for melting (see Equation (1)) is reduced. By linearly applying the reduction, it has been possible to keep a constant temperature in manufactured samples [30]. In this system, the layer thickness was 1000 μm. The samples were additively manufactured with a size of 60 × 15 × 1.5 (in mm) and mechanized to the final dimensions.

The CO_2_-SLM system used was an Aurora Labs S-Titanium Pro (Aurora Labs, Canning Vale, Australia) and it had two 150 W CO_2_ lasers that were focused on the same zone of the powder bed. In this system, the focus lens moves in the x and y axes, while the base plate moves for the deposition of each layer (z-axis). The base plate was heated to 60 °C. The SLM chamber was filled with argon, and the oxygen proportion was below 0.5% during the manufacturing process. The manufactured samples’ size was 40 × 3 ×1.5 × (in mm).

The used SLM with a fiber laser system (EOS M280 400W, EOS GmbH, Krailling, Germany) had a fiber laser and a galvanometer mirror system for the laser scan. With this method, several samples were fabricated with the YZ plane as its base plane and a rotation angle between layers of 67°. The manufactured samples’ size was 40 × 3 ×1.5 × (in mm).

**Figure 2:**
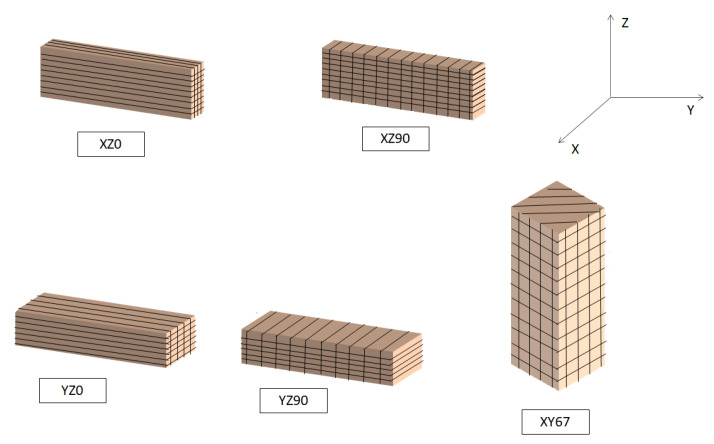
Sample orientations used.

Different kinds of samples were fabricated using the CO_2_ laser SLM. To compare them with samples made using DLD, samples in which the manufacturing base planes were XZ and XY were fabricated with angles of 0° and 90°, with an angle rotation of 0° between layers. To compare the fiber laser SLM system with the CO_2_ system, several samples were fabricated with the YZ plane as the base plane with a rotation angle of 67° between layers. In all cases, the used scan speed was 50 mm/s. The manufacturing conditions of the samples are shown in Table 2 and a representation of the angles can be observed in Figure 2.

**Figure 3:**
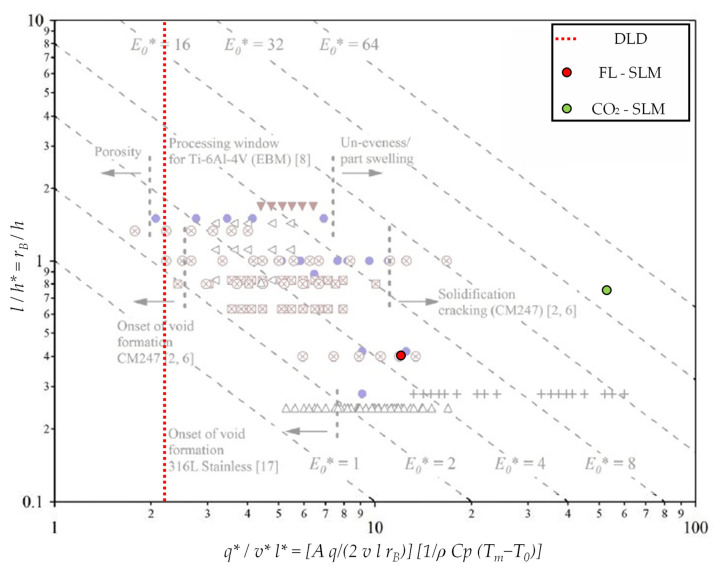
Processing diagram of several AM alloys with the materials studied in this work, based on [25]. The *x* axis is the E*_min_.

A processing diagram of the studied samples can be observed in Figure 2, in comparison to the other used SLM methods [25]. As can be seen, the parameters for the FL–SLM were similar to those of other authors as it is a well-stablished technique. For CO_2_–SLM, the required parameters for the build-up of the sample provided a higher E_min_* (*x* axis in Figure 2) to minimize the effect of the instabilities of the CO_2_ lasers. In the case of DLD, the differences in the process, particularly in terms of hatch spacing (*y* axis in Figure 3) and in layer height, make the required E_min_* smaller and *E*_0_ can be much higher.

An analysis of the quality of the manufactured samples was performed using optical microscopy and scanning electron microscopy (SEM) with a Hitachi S-3400N microscope equipped with an energy dispersive X-ray spectrometer (EDS, Bruker AXS Xflash Detector 5010, Bruker AXS Microanalysis, Berlin, Germany); samples were cut, embedded in conductive resin, mechanically polished (up to 1 mm), and electro etched in 10% oxalic acid at a current of 6 V. Porosity measurements were performed with image analysis software (Image Pro Plus) in the three directions of the samples in at least ten different zones. 

X-ray diffraction (XRD) with a Philips X’Pert diffractometer (Philips Analytical Company, Eindhoven, The Netherlands) (CuKα = 1.54056 Å) was used to identify the phases that formed in the samples.

Microhardness was determined using a microhardness tester (SHIMADZU HMV-2TE, Shimadzu, Kioto, Japan) by applying loads of 980.7 mN (HV0.1) for 15 s to the polished samples. The average hardness value was calculated after ten indentations.

**Figure 4:**
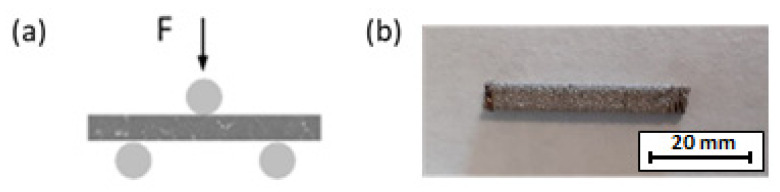
(**a**) Three-point bending test configuration; (**b**) bending sample used.

Bending samples were fabricated once the best manufacturing speed was selected after determining the porosity and microhardness results. Three-point bending tests were performed with a ZWICK/ROEL TYPE 8594.60 testing machine (ZWICK-/Roell, Ulm, Germany). The setup is shown in Figure 4a. For this test, at least three samples with dimensions of 40 × 3 × 1.5 mm^3^ were used for each manufacturing condition (Figure 4b). Bending samples were fabricated using DLD, fiber laser SLM, and CO_2_ laser SLM.

The flexural strain was calculated according to Equation (3):

(3) $$\varepsilon_{f}=\frac{6Dd}{L^{2}}$$

where *D* is the maximum deflection of the center of the substrate, *d* is the height of the sample, and *L* is the support span length. To obtain the flexural stress Equation (4) was used:

(4) $$\sigma_{f}=\frac{3L}{2bd^{2}}F$$

where *F* is the maximum load and *b* is the width of the tested sample [31,32].

The fracture surfaces obtained after the test were observed using SEM, to determine the main fracture mechanisms of the samples.

## 3. Results and Discussion

### 3.1. Microstructure

**Figure 5:**
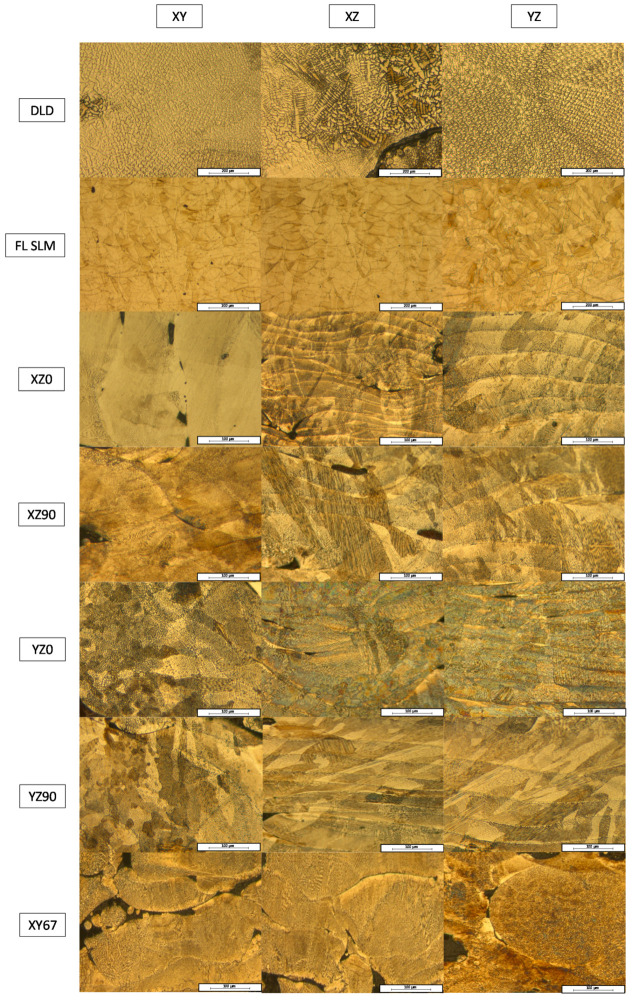
Macrostructure of the manufactured samples. Rows correspond to a manufacturing technique and orientation, and columns show the different cross sections of the specimens.

Figure 5 shows the microstructures of each kind of manufactured sample, according to its manufacturing process, its base plane, and its infill conditions.

In DLD and SLM, the samples were formed by the addition of layers to previous ones, and this had a strong effect on the microstructure of the samples. At low magnification, the structure of the layers was observed in the YZ and XZ planes.

In the DLD samples (Figure 5, third row), the layers were thicker than those in the SLM cases. Within the layers, there was no change in the microstructure with the plane. In samples made via fiber laser SLM (Figure 5, second row), a small melt pool structure in the parallel planes of the build direction was formed. The rotation angle between layers made the x and y directions equivalent. On the top, different laser scanning directions could be observed because of the rotation of the laser scanning.

In the CO_2_ SLM samples, two strategies were used to approximate the manufacturing to that of DLD geometry in the perpendicular direction, and to reproduce the pattern of the FL–SLM samples. In the former cases, the layer structure strongly depended on the base plane and the scan direction. In this case, the three planes showed clear differences. There was a plane in which the layers were elongated following the normal direction. In another, there was a structure of short melt pools; this plane corresponded to the direction perpendicular to the laser path. In the last plane, the top one, there was a structure in which the melt pools mixed, depending on the depth of the laser treatment. These effects were similar in samples XZ0, XZ90, YZ0, and YZ90 (Figure 5, rows 4–6), although the corresponding planes rotated depending on the infill and the base plane. The direction of the melt pools rotated 90° between XZ0 (Figure 5, third row) and ZX90 (Figure 5, fifth row) due to the change in the infill; and between XZ0 (Figure 5, third row) and XY0 (Figure 5, fifth row), which had similar structures in different planes.

In the case of sample XY67 (Figure 5, seventh row), the same melt pools could be seen in both lateral planes, not elongated, but rather rounded, because of the rotation angle (67° between layers), making both planes the same. This was the same case that presented with pieces made using fiber laser SLM since the scan conditions were the same. In the top plane, the same structure could be seen, which was observed in the other SLM structures. In the case of sample XY67, the same melt pools in both lateral planes can be seen, which were not elongated, and were rounded, because of the rotation angle (67° between layers), making both planes the same. This is the same case that presents with pieces made using fiber laser SLM since the scan conditions are the same. In the top plane, the same structure that is observed in the other SLM structures can be seen.

**Figure 6:**
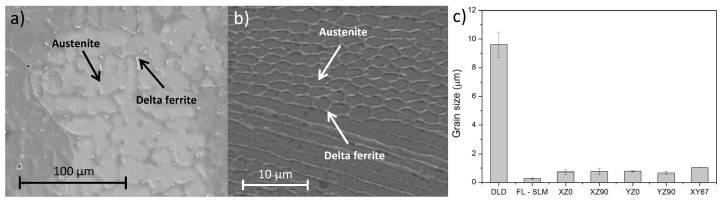
SEM micrograph of samples: (**a**) DLD; (**b**) FL–SLM; and (**c**) grain sizes measured in the samples.

At high magnifications (Figure 6), two kinds of phases can be observed in all the samples. In the case of DLD (Figure 6a), austenite with a dendritic geometry can be observed with a large interdendritic phase, which is commonly associated with delta ferrite [33,34]. In the case of the SLM samples, two different shapes of austenitic cellular grains can be appreciated. Both corresponded to the same elongated structures, but appeared as elongated or equiaxial polygons depending on the direction of the section made with the grains. The longest direction of the grains was perpendicular to the local fusion line (Figure 6b), which is the direction that had the fastest cooling. In the DLD samples, there was only one normal direction of the fusion line, and, for this reason, only one grain shape is observed. In addition, it is known that the temperature gradient at the liquid/solid interface (*G*), and the ratio of cooling rate/thermal gradient (*R*) define the final solidified microstructure [3]. In the DLD samples, the *G*/*R* ratio was lower than in SLM samples and, for this reason, dendritic morphologies could be observed.

In all cases, austenite was the predominant phase and delta ferrite was the minor one. The austenitic phase was composed of iron with gamma stabilizer elements, such as nickel and nitrogen. The ferritic phase was in the grain boundaries and was formed by alpha stabilizer elements, such as chromium and molybdenum. These elements are pushed from the liquid during the solidification and get trapped at interfaces [35], forming the delta ferrite phase [36]. Austenite has a cellular microstructure and the columnar structure corresponded to the directional cooling of the molten steel through the solid underneath [37]. Delta ferrite may have a lathy and vermicular morphology [38].

All the microstructural features are coarser in the case of the DLD pieces. Figure 6c shows that the grain size of the DLD was one order of magnitude greater than in the SLM samples. In addition, the grain size of the CO_2_–SLM samples was greater (about three times) than those of the FL–SLM ones. These behaviors can be explained by using the normalized minimum energy supplied (E_min_*) shown in Figure 3. In the case of the CO_2_–SLM samples (green circle), the provided energy tripled the value of the FL–SLM (red circle). Additionally, the normalized height was smaller, which indicates that greater energy was provided (greater *E*_0_).

**Figure 7:**
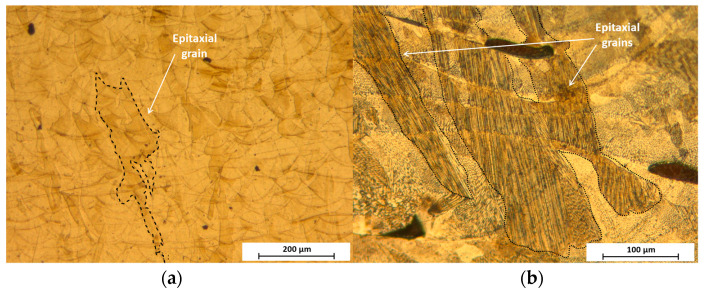
Optical micrograph: (**a**) FL–SLM sample; and (**b**) CO_2_–SLM sample.

In the case of DLD, the normalized minimum energy supplied (E_min_*) was lower than in the SLM cases; however, it was difficult to incorporate a criterion for normalized height as there was no hatch spacing. In Figure 2, this would correspond with infinity, and drives an *E*_0_ infinity value. This clearly shows the need of reducing power during manufacturing and explains the origin of the greater grain size in the DLD samples. In the FL–SLM structures (Figure 7a) the presence of epitaxial grains can be observed, which grow from the existing layers following the maximum temperature gradient direction in the building direction [39]. These epitaxial grains are composed of columnar grains, while, especially in the final layers the presence of equiaxial grains can be observed [40]. Finally, it can be observed that the grains of the CO_2_–SLM pieces (Figure 7b) are larger than in FL–SLM.

**Figure 8:**
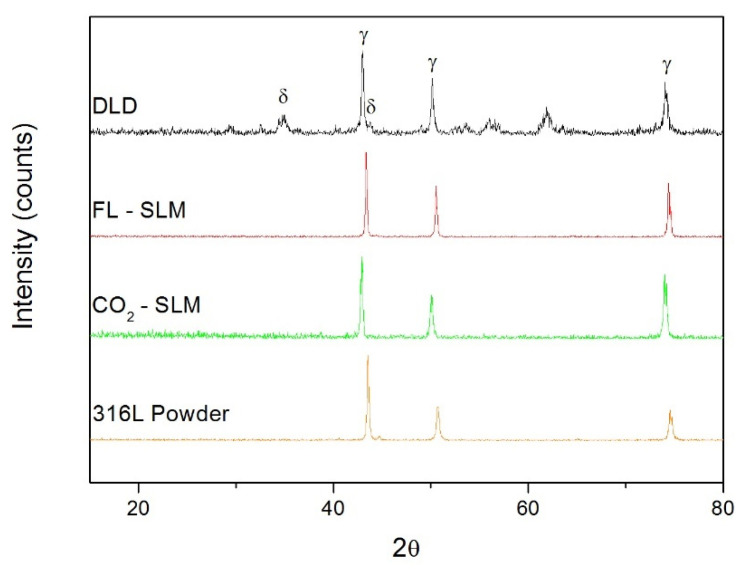
XRD of the samples made using different processes.

**Figure 9:**
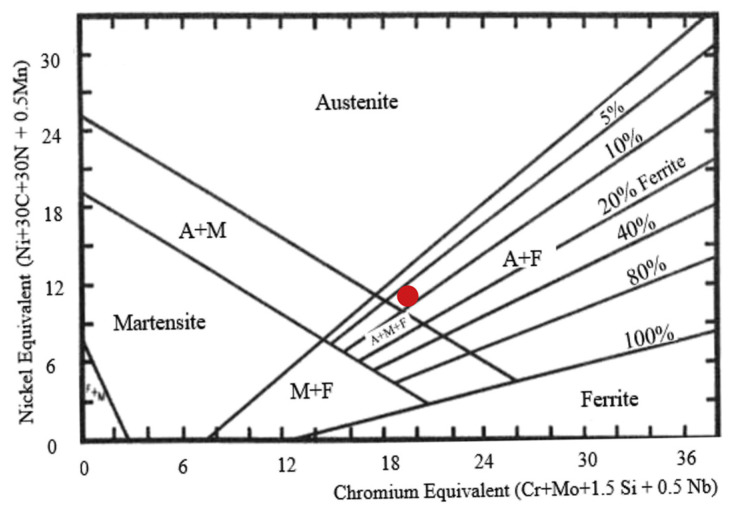
Schaeffler diagram with the 316L point marked [40].

The XRD pattern results (Figure 8) show the presence of a dual-phase formed by austenite (peaks at 43°, 52°, and 73°) and delta ferrite (peaks at 35°, 45°, and 65°) in the DLD samples but it was not detected in the SLM samples. This confirmed the SEM observations that suggested that the proportion of ferrite was greater in the DLD samples. On the other hand, in the case of samples made using DLD, there were peaks which can correspond to iron oxides enriched in Si [41]. These oxides can be caused at the end of the fabrication of these parts. A Schaeffler diagram (Figure 8) was used to predict the microstructural phases of stainless steel from a chromium equivalent (%Cr_eq_) and a nickel equivalent (%Ni_eq_) when the cooling was not in full equilibrium, as was the case of AM and welding [42]. For 316L (red mark in Figure 9), austenite with a proportion of 5–10% of delta ferrite was expected, which coincided with other studies [43].

The differences observed in the microstructures were associated with the differences in the cooling speeds of the AM processes. The general mechanism is based on the solidification of the molten pool in austenite dendrites with the direction of the cooling. In the interdendritic zones, there was a delta ferritic phase as a secondary phase [44,45]. In DLD, the amount of supplied molten material was much higher than in SLM, so the time required to cool was much longer, resulting in a lower cooling speed. Apart from the quantity of the phase formed, at a low cooling speed austenite and vermicular ferrite were formed, at medium cooling speeds austenite and interdendritic ferrite were observed, and at fast cooling speed, cellular austenite and delta ferrite were observed at grain boundaries. Although high speed in solidification can avoid the formation of delta ferrite, even in the parts made using SLM, in which the cooling is faster, this phase was found during microstructural analysis.

It has been reported that, in welding, the presence of a 5–10% delta ferrite phase improves the behavior of austenitic steels, but values above 10% reduce ductility, toughness, and corrosion resistance, while values below 5% can cause solidification cracking [46]. Therefore, its presence could degrade the properties of our samples, particularly that of the DLD ones.

### 3.2. Porosity and Defects

**Figure 10:**
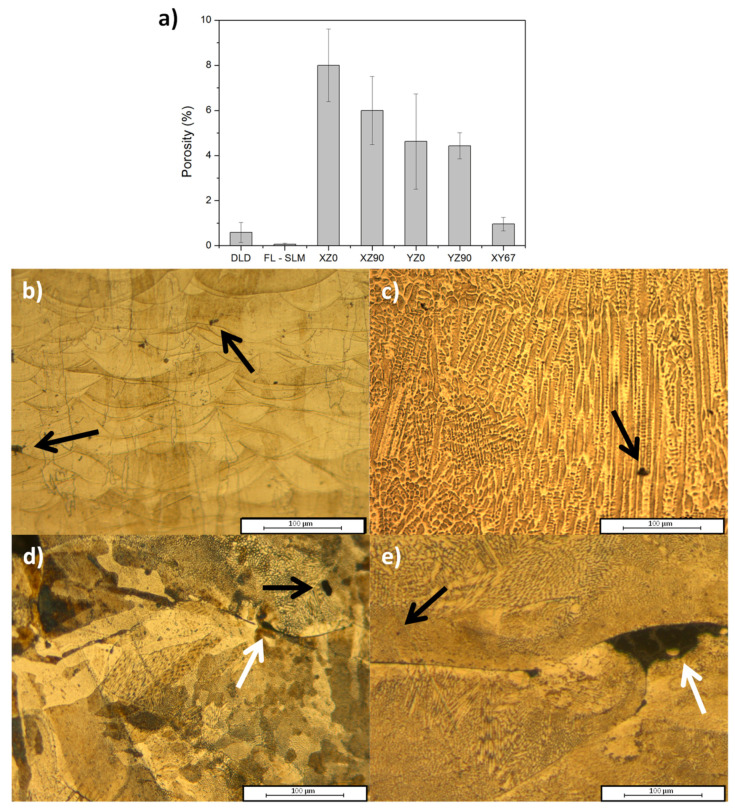
(**a**) Porosity of the samples manufactured. Pores (black arrow) and lack of fusion (white arrow) in (**b**) DLD; (**c**) FL–SLM; (**d**) CO_2_ laser XZ90 sample; (**e**) XY67 sample.

The AM technique and building strategy used caused differences in the proportion (Figure 10a), geometry, and size of the porosity of samples. The porosity in the DLD samples was 0.5%, which was a good value. The microstructure showed the presence of a reduced number of spherical pores with sizes of ~2 μm (Figure 10b). This geometry indicated that, during cooling, some gas was trapped in the molten pool [47], either from the argon used for the spraying of the powder or associated with some volatile generated during manufacturing. The thickness of the DLD layers was in the mm range, which was much higher than with other techniques. This limited the ability of the gas to escape from the molten pool and caused the appearance of this porosity.

In the fiber laser SLM samples, the porosity was very low (0.2%) and they were in the shape of small spheres (Figure 10c), indicating that they were caused by gas entrapped during the solidification of the molten pool. The small thickness of the layers (50 μm) favored the escape of gas from the liquid phase and avoided the appearance of bigger pores. The high absorption of the laser beam, along with the long depth of focus of the system used, reduced the appearance of defects caused by a lack of fusion, while the homogeneous laser spot favored a stable molten pool with a low agitation that reduced the trapping of gas.

In the SLM samples manufactured using a CO_2_ laser, greater porosity was observed (Figure 10a). Two types of defects were observed: porosity (Figure 10d–e, black arrow) and lack fusion (Figure 10d–e, white arrow). The overall porosity decreased substantially for the different strategies used: XZ0 had the highest porosity; it was reduced in XZ90; in the YZ orientation, the porosity was lower and did not depend on the infill orientation; and for XY67, i.e., in the orientation in which the laser was rotated as in the FL–SLM one (with a porosity of 0.2%), the porosity was lowest, with a value of ~1%.

The differences observed between the fiber laser SLM and the CO_2_ SLM were associated with the laser characteristics of CO_2_ radiation. The absorbance of the 10.6 μm radiation was smaller than in shorter wavelengths and the laser beam had a lower quality and the depth of focus was smaller. This caused the absorption of energy to be lower and more sensitive to variations in the powder size distribution and the homogeneity of the deposited layer. The presence of porosity with elongated and irregular geometries (Figure 10d–e, white arrow) was indicative of a lack of fusion [48]. This defect was related to the low energy absorbed by the powder which causes some powder to not melt, so it stays inside the material without consolidating with the rest of the material [49].

In addition, the infill orientation resulted as being relevant for the reduction of the porosity. Scanning the laser along the largest direction of the samples caused higher porosity in all studied cases; this geometry was the configuration that most resembled the DLD configuration, in which the molten pool is more continuous and was more prone to trapping gas. In this orientation, as well as in the perpendicular one, the repetition of the same angle in the infill favored the localization of defects. Some authors found low scan pattern angles since the overlap area between the previous and the new layer are bigger and this helps to close previous porosity and to liberate some occlude gases [50] as happened in the XY samples, which showed a lower porosity than the XZ ones. By applying a rotation angle of 67° between layers, every layer was scanned at a different angle and the zones that were prone to defects were different in each layer (Figure 10e). This strongly reduced the porosity to values below 1%.

Apart from the buildup strategy used, the presence of porosity and defects might be intrinsic to the use of CO_2_ lasers. It was very important to consider that CO_2_ lasers have an intrinsic instability in the beam as a result of how the laser beam is formed and in the way the laser light is transmitted or guided to melt powders. On the one hand, in gas lasers, there may be a relatively non-stable output of power because of the thermal expansion and contraction of the laser structure due to the heat generated in the process of pumping energy to a large volume of gas, as with CO_2_ in our case. In addition, the presence of gas turbulence in the gas-assisted heat diffusion process may induce more instability in the beam. Finally, during the emission of gas lasers, is it common to have instabilities in the polarization of the emitted laser light, which rotates with time. As the laser light is transferred by reflection off many mirrors, and due to the possible relative incidence angles between the laser light and the used powder, the reflectance of the laser light may be affected by the rotation of the polarization. All these thermal problems cannot be avoided in our system, as the laser must be switched on and off at least once per layer, so it never achieves a steady state.

In addition, CO_2_ lasers emit at 10.6 μm, which is in the medium infrared spectrum. As there are no optical fibers to guide the light, it is driven to the AM powder by space bulk reflective optics, which makes it so that the laser beam is exposed to air fluctuations in the SLM chamber. As a result, beam use is intrinsically much more unstable, which results in the presence of more defects in additively manufactured samples, and also implies the need for higher laser powers than with other laser sources. In our case, the used E*_0_ was ~32 while in the FL–SLM the E*_0_ was ~5. Thus, even with an excess of energy, a lack of fusion could not be avoided.

In the case of FL–SLM, the light provided by the laser source is stable. The emission is in the near-infrared and is transmitted by fiber, so it only travels a short length in the chamber before irradiating the metal powder. For the conditions used, the molten pool was also stable, which resulted in defect-free samples, in which only minor pores caused by the trapping of gas could be observed. The E*_0_ used was ~5, which is within the range used for most metals [25].

Finally, in DLD, the used HPDL was stable, and the heat provided was high enough to fully melt all the material deposited, which minimized the presence of a lack of fusion, resulted in the homogenization of the deposited layer, and allowed the diffusion of trapped gas out of the molten layer that was deposited. Although, as shown in Figure 3, the onset of porosity can be a problem in the working range of the DLD system, no considerable defects were found.

### 3.3. Mechanical Properties

#### 3.3.1. Microhardness

**Figure 11:**
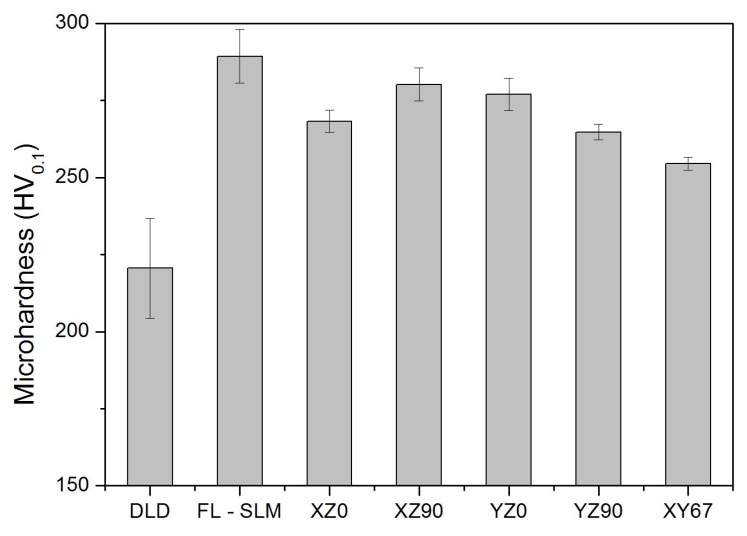
Microhardness (HV_0.1_) of the AM samples.

Microhardness results (Figure 11) showed that DLD produced samples with a Vickers hardness of 221 ± 16 HV. This value was well above the 145–155 MPa that is the standard value of bulk AISI 316L stainless steel [51] and was roughly higher than the maximum value expected for this material, which is 217 HV [52]. SLM provided higher values, with 289 ± 9 MPa for the FL–SLM and an average of 268 ± 11 HV for the different CO_2_–SLM samples. The differences observed between the systems were mainly related to the cooling time of the processes involved and the presence of defects in the samples.

In DLD, the amount of molten material per layer was very high, as the layers were thicker, and the laser spot was wider. Therefore, the time required to solidify and cool down the deposited material was much longer than in SLM. In addition, most of the cooling is made by conduction through the previously manufactured material, which is heated several times. Consequently, grains grew, resulting in a lower hardness. In addition, in the DLD samples, the presence of ferrite at grain borders was observed, which can also reduce the hardness of samples.

Hardness values are similar from SLM with a fiber laser and SLM with a CO_2_ laser, although the fiber laser produced slightly higher values. In the CO_2_–SLM samples, the scan speed was slower than in the fiber laser one, and this implied higher energy input values and higher temperatures. Therefore, as there was more heat to evacuate, cooling was slower, and it favored the formation of delta ferrite [53] and grain growth [54,55]. The CO_2_–SLM samples showed greater porosity than other samples, which also resulted in a lower hardness value because of the reduced effective density of the material. Although the CO_2_–SLM system worked in the range of energy in which pores should not appear (E*_0_ ~ 32, Figure 3), the printed parts had a high level of porosity, which indicated that ag greater energy input would be needed to fabricate full dense pieces, which would improve properties like hardness. On the other hand, the instability of this type of laser can be a reason that justifies the presence of this kind of defect, despite the energy used.

In the CO_2_–SLM samples, the build orientation and infill did not have much influence on the hardness. In the lower scan pattern angle, a higher hardness was found, and by moving across the Y-direction the average value of hardness decreased. This could be related to a higher overlap between subsequent layers. When the lower scan pattern angle was selected, the hatch space was much more coincident with the former layer. In this case, the overlap area of the second layer was mostly sitting on the first layer and two further laser crossings occurred; therefore, this leads to double remelting on the overlap of the former layers and enhanced the possible removal of keyholes and pores.

Finally, in the case of samples with a scanning rotation of 67° between layers, the hardness declined because the grains grew more than in the other cases, which coincided with studies by other authors [56,57]. In this case, the reduced porosity seemed to have favored heat conduction to the lower layers and induced greater heat treatment.

#### 3.3.2. Bending Tests

**Figure 12:**
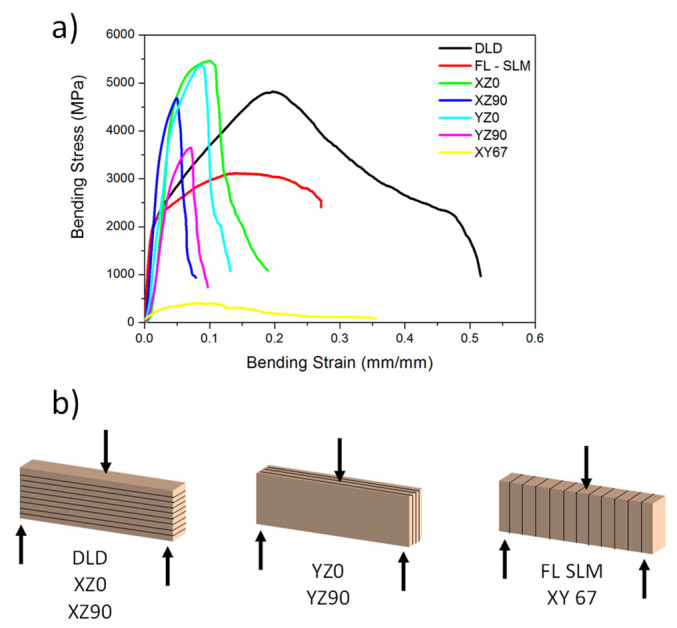
(**a**) Flexural tests of the different samples manufactured and (**b**) scheme of the flexural tests of the parts with their layers marked.

Bending tests were performed for all manufactured samples (Figure 12). There were several observed differences in the behavior of the different materials. The yield strength of DLD and FL-SLM seemed to be lower than that of the CO_2_–SLM samples, but after that point, the resistance consistently continuously increased to the maximum resistance, and, from there, decayed in a ductile way. The behavior of DLD samples showed that they had a high elongation and that the strength was very high, close to the best of the other systems. The FL–SLM showed a similar behavior, but with a lower hardening ability and with a lower fracture strain. The CO_2_–SLM samples showed higher yields and, in many cases, a higher maximum resistance; however, after its maximum, the resistance decayed abruptly, showing less fracture strain. There were differences associated with the building methodology, but all their behaviors were similar.

**Figure 13:**
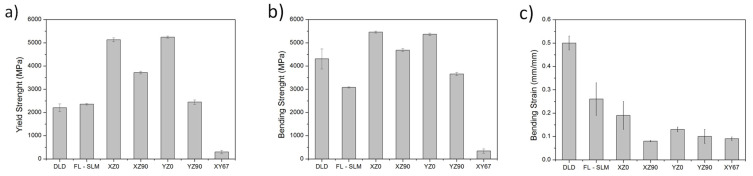
Bending test results of the AM samples: (**a**) yield strength, (**b**) maximum flexural strength, and (**c**) maximum flexural strain.

From the bending curves, the values of flexural yield, maximum strength, and maximum strain were obtained and are plotted in Figure 13. The yield of the DLD samples (Figure 13a) was smaller than that of the other systems, which was consistent with the lower hardness measured in these samples. Their resistances (Figure 13b), however, were close to the best values, while the fracture strain was the best. The fiber laser SLM samples showed a low yield and low resistance values compared with other SLM samples, but they had a good maximum flexural strain.

The CO_2_–SLM samples showed different behaviors depending on the infill orientation. The yield (Figure 13a) and the strength (Figure 13b) were maximum when the samples were manufactured with the infill in the longest directions of the samples (XZ0 and YZ0 samples), and it was much lower when it was in the transversal one (XZ90 and YZ90) or with a rotation of 67° (XY67). The values were, in general, larger than in FL–SLM and greater or comparable to those of DLD. The maximum flexural strain measured in the samples (Figure 13c) was much larger for the DLD samples than for the rest. The CO_2_–SLM samples are the those with a lower fracture strain, and no significant effect of the building orientation or infill direction is observed. The DLD samples had 178% more fracture strain than the best CO_2_ sample (XZ0) and 92% more fracture strain than the FL–SLM samples.

The observed results are very dependent on the microstructure of the samples. The DLD samples had a coarse grain structure. Large grains are associated with an increase in plastic deformation ability because the dislocations have more freedom to move since the grain borders do not act as obstacles. These characteristics favor having high fracture strain values, but not high yield values. In addition, the DLD samples were manufactured along the length direction, so there were no discontinuities in the transversal direction of the samples that would have caused premature failure. Finally, there was a low presence of pores and defects in the samples, so there were also no stress concentration zones. All these features explain the high fracture strain of these samples and the low yield. Despite this, the deformation of the material during bending causes strain hardening, which increases the maximum resistance observed in these samples. The presence of ferrite may have limited the maximum bending strength and the fracture strength since it is a brittle phase that appears in the studied cases in the grain borders. Other authors claim that this ferrite can act as a dislocation barrier, which confirms that the mechanical properties can be limited [58].

The SLM samples showed a fine microstructure with small grains in the transversal section and larger ones along the surface. This causes different behavior in terms of hardness and mechanical testing. The results showed that there was a strong effect of infill orientation in the results of the tests. The maximum strength was enhanced when the infill was in the lengthwise direction of the sample, a condition that is similar to that of the DLD samples. Additionally, the orientation in which the sample is built is important. Samples in which the base plane is XY have a greater ability to support more loads. In this build orientation, the laser path that is traveled to build the sample is shorter, resulting in a greater concentration of heat, which helps to reduce defects like porosity and improve the melting of the whole structure. On the other hand, structures where the base plane is XZ are built with a longer travel of the laser, so heat is less concentrated, and faster cooling takes place, which causes the grain size to decrease. This effect reduces fracture strain and improves strength.

In SLM samples, for both the fiber laser and CO_2_ laser, grains are smaller than in DLD and the number of grain boundaries is higher. Therefore, dislocations find more obstacles to move, and plastic deformation is less favored than in DLD. Figure 12a shows that the yield of the CO_2_ –SLM is much higher than that of DLD samples, but it breaks at low strain values, presumably due to the presence of defects that act as stress concentration points.

Remarkably, most of the CO_2_–SLM samples show higher strength than the fiber laser–SLM samples. This can be explained through the orientation of the laser scan. In the fiber laser samples, the build-up is made with rotations of 67° of the laser scans and this causes a global homogeneous behavior that has local defects in the sense of having a microstructure in which long grains and short ones are simultaneously strained. This behavior is similar to that of the XY67 sample manufactured using the CO_2_ laser, which showed the worst resistance values (Figure 13b).

In the 0° infill, sample strength decreases due to the long distance the laser travels that allows the heat to accumulate, the 90° infill samples had increased strength because their layers consolidated better, and the short laser distances did not have an affect like in the 0° samples. Finally, the samples built on the XY plane had the biggest tendency to break with low loads because the load direction was normal to the build direction, as shown in Figure 12b, which caused the crack to have a favorable growth between layers, suggesting that the bonding between layers was weaker in this direction. This confirms that, in the CO_2_–SLM samples, the presence of defects and brittle zones between layers reduced their performance when the stress was concentrated in these zones.

Residual stress can be considered to affect the mechanical properties of AM parts. Although no measurements have been made in this study, the as-built parts did not show a great deformation, which indicates that the stress was equilibrated and that it might not have had a strong influence in crack initiation.

On the other hand, it can be observed that the build orientation has little influence on the ability of deformation of each kind of sample (Figure 13c) because this mechanism is dominated by grain size. Although build orientation affects parameters like laser travel and this affects the quantity of energy that each layer receives and how it melts, cooling is not affected by this parameter much, so the results, in this case, are very similar.

#### 3.3.3. Fracture Tests

**Figure 14:**
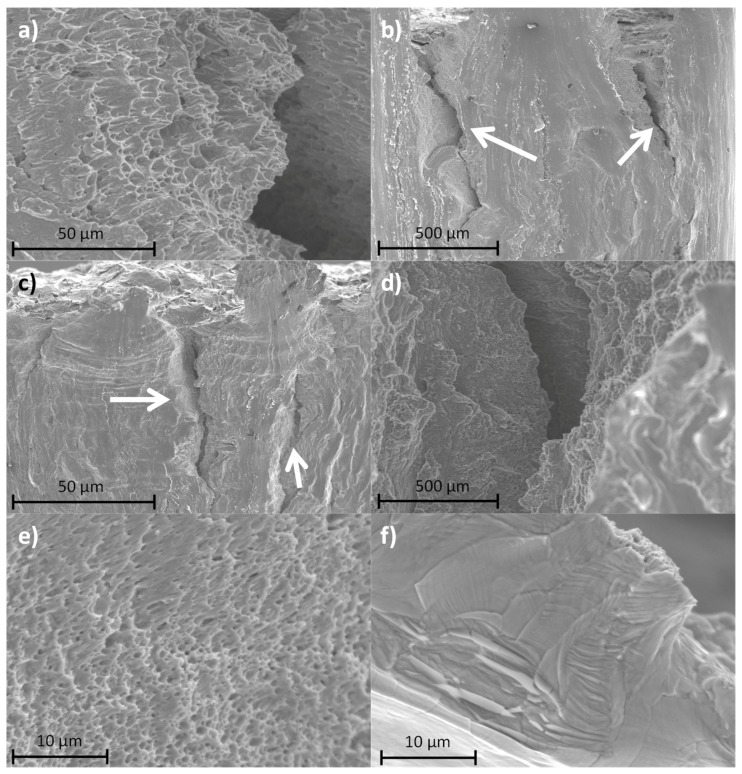
Fracture surfaces: (**a**) DLD; (**b**) zone of DLD with brittle fractures; (**c**) FL–SLM and (**d**) detail of FL–SLM; (**e**) and (**f**) YZ0 CO_2_ laser SLM.

Figure 14 shows the fractography of the samples after the bending test. Figure 13a,b shows the fractography of the DLD samples. The images show that the dominating behavior was ductile fracture (Figure 14a) with characteristic dimples that evidenced the presence of a high strain before breaking and zones with a low roughness. However, other zones showed discontinuities in this behavior; Figure 14b shows that the fracture occurs in different planes. On the other hand, the presence of secondary cracks can be seen, favored by the presence of secondary phases like delta ferrite, which can provoke nucleation of cracks. These results coincide with other studies on DED techniques [21].

SLM samples presented a fracture surface composed of cleavage features with little dimples (Figure 14c,d), which coincided with other studies [59]. Compared with the DLD technique, a more fragile fracture due to its faster cooling is found, which provokes less ductility, as shown previously. 

CO_2_ laser SLM fractography is shown in Figure 14e,f. There is a dense structure of dimples formed in the fractures, but their size is significantly smaller than in the case of the DLD samples, and are comparable with those of the parts manufactured using a fiber laser. The presence of unmelted powder particles seems to have initiated cracks, as in other systems [60].

**Figure 15:**
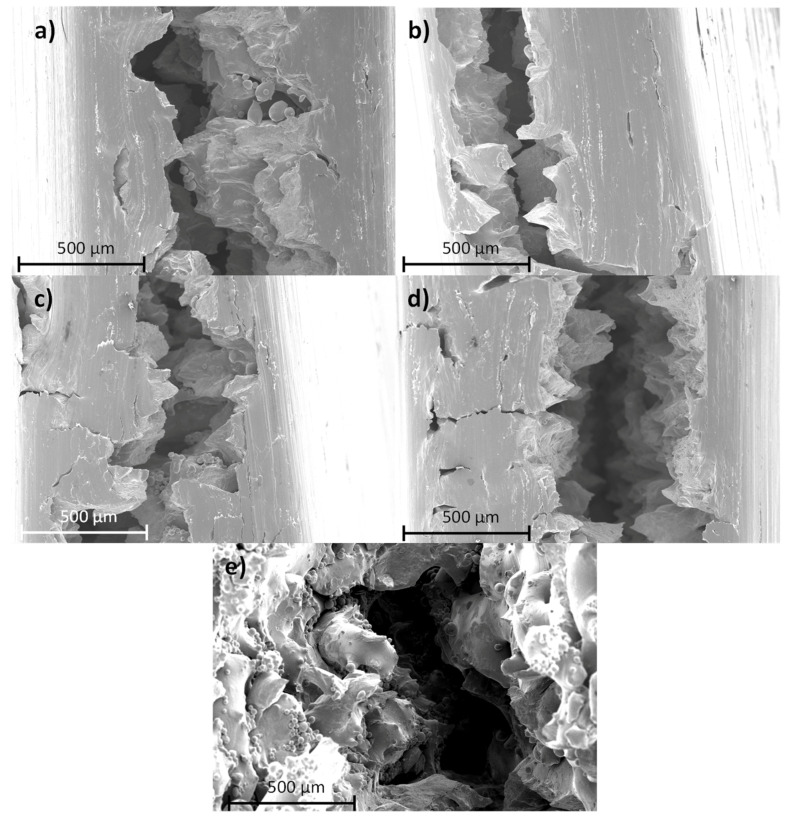
Fracture surfaces of CO_2_ laser SLM samples: (**a**) XZ0; (**b**) XZ90; (**c**) YZ0; (**d**) YZ90; and (**e**) XY67.

On the other hand, infill did not provoke any different fractures, which explains the similar values in the mechanical properties when the other conditions were the same. However, the built direction can change fracture morphology, as shown in Figure 15. In the XY base plane samples (Figure 15a,b), each layer broke individually, the crack grew to break, one by one; in the XZ (Figure 15c,d) base plane samples, every layer broke at the same time, and the crack grew through them. This means that, in the XZ base plane samples, the fracture morphology repeated along the thickness of the sample and in the YZ base plane samples it can be observed that the fractured structure was not repeated layer by layer.

## 4. Conclusions

We fabricated 316L stainless steel samples using DLD, fiber laser SLM, and CO_2_ laser SLM using different manufacturing build directions and rotation angles between layers. The main conclusions that were obtained are the following:

(1) All AM technologies used provide austenitic grains with delta ferrite in the border grains. This last phase is larger in the DLD samples, and its grains are the largest.
(2) Porosity is smaller in the pieces made using DLD and fiber laser SLM. The samples made using CO_2_ laser SLM have a larger porosity, which depends on the manufacturing parameters, but values below 1% were achieved by rotating the infill laser treatment.
(3) The hardest sample is the one made using fiber laser SLM, while the DLD samples present low hardness. Pieces made using the CO_2_ laser SLM present intermediate values, which are 8% softer than the those of the fiber SLM pieces and 17% harder than those made using DLD.
(4) CO_2_ laser SLM provides high strength and yield strength in flexural testing, higher than DLD and FL-SLM in some used manufacturing strategies because of the prepared layer structure to support the loads and because of the fine grains. However, they have a much lower fracture strain than other techniques. This is mainly due to the presence of defects in the microstructure.
(5) The fractures that the samples present are ductile. Unmelted particles can be seen, which provoke porosity and this defect can produce fractures by pore coalescence. The morphology of the fracture is very much influenced by the layer structure of the samples.
(6) The differences in properties have been explained with the help of normalized parameters that has allowed comparing and explaining the observed results despite the differences shown in the techniques used. The results indicate that despite using a higher normalized equivalent energy density, porosity and other defects still appear, which can be explained by the intrinsic instabilities of the CO_2_ laser.

## Figures and Tables

**Table 1:** Characteristics of the additive manufacturing systems used.

	DLD	FL-SLM	CO_2_-SLM
Laser type	Diode CW	Fiber CW	CO_2_ CW
Spot shape	Rectangular	Gaussian	Gaussian
Laser spot size (mm)	1.25 × 0.6	0.070 ∅	0.150 ∅
V scan (mm/s)	10	1083	50
Laser Power (W)	600–100	195	255
Layer height (μm)	1000	20	60
λ (nm)	810	1070	10,600
Intensity (W/cm^2^)	8 × 10^4^	5 × 10^6^	1.45 × 10^3^
Absorptivity	0.36	0.53	0.53

**Table 2:** Parameters and conditions used in the additive manufacturing processes.

Sample	Technique	Base Plane	Infill Orientation
DLD	DLD	XZ	0
FL-SLM	Fiber laser SLM	XY	67° btw layers
XZ0	CO_2_ laser SLM	XZ	0
XZ90	CO_2_ laser SLM	XZ	90
YZ0	CO_2_ laser SLM	YZ	0
YZ90	CO_2_ laser SLM	YZ	90
XY67	CO_2_ laser SLM	XY	67° btw layers

## References

[1] Milewski J.O. (2017). Additive Manufacturing of Metals.

[2] Yang L., Hsu K., Baughman B., Godfrey D., Medina F., Menon M., Wiener S. (2017). Additive Manufacturing of Metals: The Technology, Materials, Design and Production.

[3] DebRoy T., Wei H.L., Zuback J.S., Mukherjee T., Elmer J.W., Milewski J.O., Beese A.M., Wilson-Heid A., De A., Zhang W. (2018). Additive manufacturing of metallic components—Process, structure and properties. Prog. Mater. Sci..

[4] Malekipour E., El-Mounayri H. (2018). Common defects and contributing parameters in powder bed fusion AM process and their classification for online monitoring and control: A review. Int. J. Adv. Manuf. Technol..

[5] du Plessis A., Yadroitsava I., Yadroitsev I. (2020). Effects of defects on mechanical properties in metal additive manufacturing: A review focusing on X-ray tomography insights. Mater. Des..

[6] Cooke S., Ahmadi K., Willerth S., Herring R. (2020). Metal additive manufacturing: Technology, metallurgy and modelling. J. Manuf. Process..

[7] Yap C.Y., Chua C.K., Dong Z., Liu Z.H., Zhang D.Q., Loh L.E., Sing S.L. (2015). Review of selective laser melting: Materials and applications. Appl. Phys. Rev..

[8] Riquelme A., Rodrigo P., Escalera-Rodriguez M., Rams J. (2019). Effect of the process parameters in the additive manufacturing of in situ Al/AlN samples. J. Manuf. Process..

[9] Thompson S.M., Bian L., Shamsaei N., Yadollahi A. (2015). An overview of Direct Laser Deposition for additive manufacturing; Part I: Transport phenomena, modeling and diagnostics. Addit. Manuf..

[10] Shamsaei N., Yadollahi A., Bian L., Thompson S.M. (2015). An overview of Direct Laser Deposition for additive manufacturing; Part II: Mechanical behavior, process parameter optimization and control. Addit. Manuf..

[11] Zhu H., Fu X., Fan S., Liang L., Lin X., Ning Y. (2020). The conversion from a Gaussian-like beam to a flat-top beam in the laser hardening processing using a fiber coupled diode laser source. Opt. Laser Technol..

[12] Lee H., Lim C.H.J., Low M.J., Tham N., Murukeshan V.M., Kim Y.J. (2017). Lasers in additive manufacturing: A review. Int. J. Precis. Eng. Manuf. Technol..

[13] Prabakaran M., Kannan G. (2018). Optimization of CO2 Laser Beam Welding Process Parameters to Attain Maximum Weld Strength in Dissimilar Metals. Mater. Today Proc..

[14] Sander G., Thomas S., Cruz V., Jurg M., Birbilis N., Gao X., Brameld M., Hutchinson C.R. (2017). On The Corrosion and Metastable Pitting Characteristics of 316L Stainless Steel Produced by Selective Laser Melting. J. Electrochem. Soc..

[15] Feng Y., Luo Z., Liu Z., Li Y., Luo Y., Huang Y. (2015). Keyhole gas tungsten arc welding of AISI 316L stainless steel. Mater. Des..

[16] Kianersi D., Mostafaei A., Amadeh A.A. (2014). Resistance spot welding joints of AISI 316L austenitic stainless steel sheets: Phase transformations, mechanical properties and microstructure characterizations. Mater. Des..

[17] Kong D., Ni X., Dong C., Lei X., Zhang L., Man C., Yao J., Cheng X., Li X. (2018). Bio-functional and anti-corrosive 3D printing 316L stainless steel fabricated by selective laser melting. Mater. Des..

[18] Chen X., Li J., Cheng X., He B., Wang H., Huang Z. (2017). Microstructure and mechanical properties of the austenitic stainless steel 316L fabricated by gas metal arc additive manufacturing. Mater. Sci. Eng. A.

[19] Gong H., Snelling D., Kardel K., Carrano A. (2019). Comparison of Stainless Steel 316L Parts Made by FDM- and SLM-Based Additive Manufacturing Processes. JOM.

[20] Bartolomeu F., Buciumeanu M., Pinto E., Alves N., Carvalho O., Silva F., Miranda G. (2017). 316L stainless steel mechanical and tribological behavior—A comparison between selective laser melting, hot pressing and conventional casting. Addit. Manuf..

[21] Barkia B., Aubry P., Haghi-Ashtiani P., Auger T., Gosmain L., Schuster F., Maskrot H. (2020). On the origin of the high tensile strength and ductility of additively manufactured 316L stainless steel: Multiscale investigation. J. Mater. Sci. Technol..

[22] Trelewicz J.R., Halada G.P., Donaldson O.K., Manogharan G. (2016). Microstructure and Corrosion Resistance of Laser Additively Manufactured 316L Stainless Steel. JOM.

[23] Margerit P., Weisz-Patrault D., Ravi-Chandar K., Constantinescu A. (2021). Tensile and ductile fracture properties of as-printed 316L stainless steel thin walls obtained by directed energy deposition. Addit. Manuf..

[24] Zhao C., Bai Y., Zhang Y., Wang X., Xue J.M., Wang H. (2021). Influence of scanning strategy and building direction on microstructure and corrosion behaviour of selective laser melted 316L stainless steel. Mater. Des..

[25] Thomas M., Baxter G.J., Todd I. (2016). Normalised model-based processing diagrams for additive layer manufacture of engineering alloys. Acta Mater..

[26] Kale A.B., Kim B.-K., Kim D.-I., Castle E., Reece M., Choi S.-H. (2020). An investigation of the corrosion behavior of 316L stainless steel fabricated by SLM and SPS techniques. Mater. Charact..

[27] Kamath C., Eldasher B.S., Gallegos G.F., King W.E., Sisto A. (2014). Density of additively-manufactured, 316L SS parts using laser powder-bed fusion at powers up to 400 W. Int. J. Adv. Manuf. Technol..

[28] Ziółkowski G., Chlebus E., Szymczyk-Ziółkowska P., Kurzac J. (2014). Application of X-ray CT method for discontinuity and porosity detection in 316L stainless steel parts produced with SLM technology. Arch. Civ. Mech. Eng..

[29] Riquelme A., Rodrigo P., Escalera-Rodríguez M., Rams J. (2020). Additively Manufactured Al/SiC Cylindrical Structures by Laser Metal Deposition. Materials.

[30] Riquelme A., de Rojas Candela C.S., Rodrigo P., Rams J. (2022). Influence of process parameters in additive manufacturing of highly reinforced 316L / SiCp composites. J. Mater. Process. Technol..

[31] Documents R., Factor S., Toughness P.F. (1997). Standard Test Method for Plane-Strain Fracture Toughness of Metallic Materials 1. Configurations.

[32] Pulido-González N., Torres B., Rodrigo P., Hort N., Rams J. (2020). Microstructural, mechanical and corrosion characterization of an as-cast Mg–3Zn–0.4Ca alloy for biomedical applications. J. Magnes. Alloy..

[33] Wang Y.M., Voisin T., McKeown J., Ye J., Calta N., Li Z., Zeng Z., Zhang Y., Chen W., Roehling T.T. (2018). Additively manufactured hierarchical stainless steels with high strength and ductility. Nat. Mater..

[34] Kurian S., Mirzaeifar R. (2020). Deformation mechanisms of the subgranular cellular structures in selective laser melted 316L stainless steel. Mech. Mater..

[35] Prashanth K., Eckert J. (2017). Formation of metastable cellular microstructures in selective laser melted alloys. J. Alloys Compd..

[36] Aversa A., Saboori A., Librera E., de Chirico M., Biamino S., Lombardi M., Fino P. (2020). The role of Directed Energy Deposition atmosphere mode on the microstructure and mechanical properties of 316L samples. Addit. Manuf..

[37] Liying L., Jun X., Bin H., Xiaolei W. (2019). Microstructure and mechanical properties of welded joints of L415/316L bimetal composite pipe using post internal-welding process. Int. J. Press. Vessel. Pip..

[38] Alali M., Todd I., Wynne B. (2017). Through-thickness microstructure and mechanical properties of electron beam welded 20 mm thick AISI 316L austenitic stainless steel. Mater. Des..

[39] Zhai W., Zhu Z., Zhou W., Nai S.M.L., Wei J. (2020). Selective laser melting of dispersed TiC particles strengthened 316L stainless steel. Compos. Part B Eng..

[40] Saboori A., Piscopo G., Lai M., Salmi A., Biamino S. (2020). An investigation on the effect of deposition pattern on the microstructure, mechanical properties and residual stress of 316L produced by Directed Energy Deposition. Mater. Sci. Eng. A.

[41] Yáñez S., Sánchez Andújar M., Castro-Garcia S., Mira J., Rivas J., Senaris-Rodriguez M.A. (2010). Magnetocapacitance in Fe_3_O_4_ and NiFe_2_O_4_ Nanoparticles. Boletin de la Sociedad Espanola de Ceramica y Vidrio.

[42] Schaeffler A.L. (1949). Construction Diagram for Stainless Steel Weld Metal. Metal Prog..

[43] Guo P., Zou B., Huang C., Gao H. (2017). Study on microstructure, mechanical properties and machinability of efficiently additive manufactured AISI 316L stainless steel by high-power direct laser deposition. J. Mater. Process. Technol..

[44] Ma C., Peng Q., Mei J., Han E.-H., Ke W. (2018). Microstructure and corrosion behavior of the heat affected zone of a stainless steel 308L-316L weld joint. J. Mater. Sci. Technol..

[45] Suutala N., Takalo T., Moisio T. (1980). Ferritic-austenitic solidification mode in austenitic stainless steel welds. Met. Mater. Trans. A.

[46] Kou S. (2002). Welding Metallurgy.

[47] Sames W., Medina F., Peter W., Babu S.D.R. Effect of Process Control and Powder Quality on Inconel 718 Produced Using Electron Beam Melting. Proceedings of the 8th International Symposium on Superalloy 718 and Derivatives.

[48] Darvish K., Chen Z., Pasang T. (2016). Reducing lack of fusion during selective laser melting of CoCrMo alloy: Effect of laser power on geometrical features of tracks. Mater. Des..

[49] Bauereiß A., Scharowsky T., Körner C. (2014). Defect generation and propagation mechanism during additive manufacturing by selective beam melting. J. Mater. Process. Technol..

[50] Khorasani A.M., Gibson I., Awan U.S., Ghaderi A. (2019). The effect of SLM process parameters on density, hardness, tensile strength and surface quality of Ti-6Al-4V. Addit. Manuf..

[51] Yusuf S.M., Chen Y., Boardman R., Yang S., Gao N. (2017). Investigation on Porosity and Microhardness of 316L Stainless Steel Fabricated by Selective Laser Melting. Metals.

[52] Gong Y., Yang Y., Qu S., Li P., Liang C., Zhang H. (2019). Laser energy density dependence of performance in additive/subtractive hybrid manufacturing of 316L stainless steel. Int. J. Adv. Manuf. Technol..

[53] Yang Y., Gong Y., Li C., Wen X., Sun J. (2021). Mechanical performance of 316 L stainless steel by hybrid directed energy deposition and thermal milling process. J. Mater. Process. Technol..

[54] Bang G.B., Kim W.R., Kim H.K., Park H.-K., Kim G.H., Hyun S.-K., Kwon O., Kim H.G. (2021). Effect of process parameters for selective laser melting with SUS316L on mechanical and microstructural properties with variation in chemical composition. Mater. Des..

[55] Peng T., Lv J., Majeed A., Liang X. (2021). An experimental investigation on energy-effective additive manufacturing of aluminum parts via process parameter selection. J. Clean. Prod..

[56] Mahamood R.M., Akinlabi E.T. (2017). Scanning Speed Influence on the Microstructure and Micro hardness Properties of Titanium Alloy Produced by Laser Metal Deposition Process. Mater. Today Proc..

[57] Mukherjee T., DebRoy T., Lienert T., Maloy S., Hosemann P. (2021). Spatial and temporal variation of hardness of a printed steel part. Acta Mater..

[58] Chen J., Wei H., Bao K., Zhang X., Cao Y., Peng Y., Kong J., Wang K. (2021). Dynamic mechanical properties of 316L stainless steel fabricated by an additive manufacturing process. J. Mater. Res. Technol..

[59] Yin S., Yan X., Jenkins R., Chen C., Kazasidis M., Liu M., Kuang M., Lupoi R. (2019). Hybrid additive manufacture of 316L stainless steel with cold spray and selective laser melting: Microstructure and mechanical properties. J. Mater. Process. Technol..

[60] Koutiri I., Pessard E., Peyre P., Amlou O., De Terris T. (2018). Influence of SLM process parameters on the surface finish, porosity rate and fatigue behavior of as-built Inconel 625 parts. J. Mater. Process. Technol..

